# Special Issue: Redox Active Natural Products and Their Interaction with Cellular Signalling Pathways

**DOI:** 10.3390/molecules191219588

**Published:** 2014-11-26

**Authors:** Claus Jacob

**Affiliations:** Division of Bioorganic Chemistry, School of Pharmacy, Saarland University, Campus B 2.1, D-66123 Saarbruecken, Germany; E-Mail: c.jacob@mx.uni-saarland.de; Tel.: +49-681-302-3129; Fax: +49-681-302-3464

**Keywords:** redox modulation, natural products, secondary metabolites, reactive sulfur species, catalytic sensor/effector agents, cellular thiolstat, intracellular diagnostics, nutrition, drug development, antimicrobial activity, anticancer activity

## Abstract

During the last decade, research into natural products has experienced a certain renaissance. The urgent need for more and more effective antibiotics in medicine, the demand for ecologically friendly plant protectants in agriculture, “natural” cosmetics and the issue of a sustainable and healthy nutrition in an ageing society have fuelled research into Nature’s treasure chest of “green gold”. Here, redox active secondary metabolites from plants, fungi, bacteria and other (micro-)organisms often have been at the forefront of the most interesting developments. These agents provide powerful means to interfere with many, probably most cellular signaling pathways in humans, animals and lower organisms, and therefore can be used to protect, *i.e.*, in form of antioxidants, and to frighten off or even kill, *i.e.*, in form of repellants, antibiotics, fungicides and selective, often catalytic “sensor/effector” anticancer agents. Interestingly, whilst natural product research dates back many decades, in some cases even centuries, and compounds such as allicin and various flavonoids have been investigated thoroughly in the past, it has only recently become possible to investigate their precise interactions and mode(s) of action inside living cells. Here, fluorescent staining and labelling on the one side, and appropriate detection, either qualitatively under the microscope or quantitatively in flow cytometers and plate readers, on the other, enable researchers to obtain the various pieces of information necessary to construct a fairly complete puzzle of how such compounds act and interact in living cells. Complemented by the more traditional activity assays and Western Blots, and increasingly joined by techniques such as proteomics, chemogenetic screening and mRNA profiling, these cell based bioanalytical techniques form a powerful platform for “intracellular diagnostics”. In the case of redox active compounds, especially of Reactive Sulfur Species (RSS), such techniques have recently unraveled concepts such as the “cellular thiolstat”, yet considerably more research is required in order to gain a full understanding of why and how such compounds act—often selectively—in different organisms.

For centuries, natural products obtained from plants, fungi and various (micro-)organisms have provided valuable contributions to nutrition, medicine, pharmacy, agriculture and, more recently, functional foods and anti-aging cosmetics [1]. Indeed, Nature endows us with a treasure chest full of different materials, from crude extracts, oils and distillates to ultimately pure compounds. Among these vastly different substances and products, redox active compounds are of particular interest. Such products have been around for many decades, yet only recently have witnessed a certain renaissance, encouraged by various changes in society. These changes—one may also call them developments—include: (a) an ageing process characteristic of developed societies which demands research into healthy food for the elderly (Generation 50+); (b) the demand for new ecologically friendly plant protective agents (so-called “green pesticides”) which are compatible with the environment and food chain yet also need to be cheap and locally available; (c) the emergence of multi-resistant pathogenic bacteria and fungi and hence the need for new antibiotics and antimicrobial agents; (d) the idea that certain foodstuffs may contain compounds which selectively kill cancer cells and other out-of-control cells, hence providing the key to high efficiency and selectivity, and, on a more technological side; (e) the use of modern nanotechnology to endow a whole range of hitherto “unavailable” natural products and substances with solubility/bioavailability and (f) the ability of new staining and detecting methods to follow intracellular redox and signaling processes in form of a sophisticated “intracellular diagnostics” [2,3,4].

Not surprisingly, these developments, and there are more, such as the common belief that natural cosmetics are better than artificial ones, have moved the subject of redox active secondary metabolites, their origins, production, physico-chemical properties, various biological activities and often complex modes of action right to the center of research and development. This Special Issue on redox active natural products therefore comes at a time when research in this field is gathering steam, in academia as well as in industry, and when the demand for such products is growing rapidly.

In fact, one may even talk about a new “Gold Rush” of the 21st Century, this time for the “green gold” and the various nuggets of chemical compounds hidden within it. The contributions of this Special Issue support this particular perception. Here, redox active compounds derived from common plants, such as garlic (e.g., allicin) or contained within various fruits (e.g., flavonoids, polyphenols), but also substances from more “exotic” sources, such as lichens, carnivorous plants and cacti, reflect the renewed interest in natural redox active agents [5,6,7,8,9]. Some of these materials may be readily available, such as selenium, garlic and onions, which are found or cultivated in considerable amounts worldwide. Others are more difficult to obtain and require a wider screen of plants, bacteria and fungi for biologically active components. Indeed, exotic places may hide exotic organisms containing equally exotic compounds, and the hunt is on for such materials, in the most remote places on Earth, geographically as well as conceptually. Here, Indiana Jones may well meet the Medicine Man, not only in the Amazon River region, but perhaps also in a sewer looking for promising organisms there or on the local graveyard joining Frankenstein in digging out half-rotten corpses full of maggots [10,11]. In any case, the search for new sources of natural products is also a search for new resources, scientifically as well as economically. Hence the Gold Rush metaphor may not be far-fetched. It also warns us of the consequences if trees are cut down for their barks like elephants for their ivory, fields of onions replace the natural habitat as part of new monocultures or rare plant species are ransacked and ultimately threatened by extinction.

In any case, the potential of such redox active products is beyond doubt and may be demonstrated by a few examples. In an ageing society facing demographic changes, a healthy nutrition of the wider population is pivotal to ensure a decent quality of life at an advanced age and to safeguard the health system from collapse. As oxidative stress (OS) and OS-related damages increase with age, a balanced diet rich in chemopreventive species, especially vitamins and antioxidants, seems to be of particular importance. Indeed, compounds such as cudarflavone B may well exhibit neuroprotective properties, as will be discussed in one of the papers of this Special Issue [12]. Yet the whole notion of antioxidants, if and how they may work, is controversial and requires extensive research. Not surprisingly, at least four papers of this Special Issue deal explicitly with antioxidants, including flavonoids, and show that such compounds are not simply donators of electrons or “reducing equivalents” but often interfere with complex signaling pathways, including the Nrf2-pathway, which is also a topic of several contributions [5,6,8,12,13,14,15].

More recently, redox modulating pro-oxidants, such as the thiol-reactive thiosulfinate allicin from garlic and various quinones have entered the scene. Unlike antioxidants, these compounds convey their redox signal via oxidation, for instance by modification of critical cysteine proteins and enzymes. Indeed, the ability of many natural products to modify thiol groups in proteins rather selectively has given rise to the notion of the “cellular thiolstat” [2,7,9,16]. It has also attracted the interest of pharmaceutical and agricultural research. Here, redox modulating, often catalytic “sensor/effector” agents promise an elegant approach to combine high efficiency—and hence cytotoxicity—with considerable selectivity for cells with an already disturbed redox balance and organisms particularly sensitive to redox intervention [17,18,19]. Such targets for redox modulation include, for instance, aggressively proliferating cells, but also pathogenic organisms affecting humans, animals and plants. Not surprisingly, allicin and related polysulfanes are currently under discussion in the context of cancer, inflammatory and infectious diseases and systemic sclerosis, whilst mixtures of such reactive sulfur species (RSS) are already used as green pesticides in agriculture [1,3,4,20,21,22,23,24,25,26].

Although such practical uses of natural products are often hampered by poor solubility and bioavailability, chemical modifications of such lead structures, such as the ones described in the paper on xanthenedione derivatives, but also modern nanotechnology, provide interesting answers [13,27]. By designing more soluble and/or lipophilic molecules, or by using nanoparticles, respectively, hitherto barely soluble natural products, such as hesperidin and quercetin, may become “available” for practical applications. This, by the way, also applies to the field of cosmetics, where nanotechnology is used increasingly to deliver otherwise poorly soluble antioxidants to the consumer interested in premature mummification.

Last but not least, the “Green Gold Rush” in the field of redox active natural products is not only fueled by demographic changes and emerging needs of modern society, but also by methodological advances which increasingly enable researchers to follow the mode(s) of action of such compounds inside the cell or organism, and to determine specific targets and mechanisms. This Special Issue contains several examples of such cell biological/biochemical studies which provide essential information about the action of such natural products, and often also pinpoint cellular targets, possible side effects and space for improvements (e.g., in form of chemical modification). Indeed, colorful maps of intracellular pathways, such as the one decorating the paper on redox modulating Nrf2 activators, would not be possible without a barrage of modern techniques conveniently summarized as “intracellular diagnostics” [2,15]. As part of this approach, which is discussed in detail in the paper bearing this title, fluorescent dyes indicative of certain intracellular processes, such as changes to the mitochondrial membrane potential ΔΨ_M_ or intracellular ROS, thiol or Ca^2+^ levels, are combined with fluorescently labelled antibodies in the hunt for specific proteins, and with activity assays to estimate changes to the activity of specific enzymes [3,4,23,24,28]. It is even possible to stain specific cellular organelles to “diagnose” their particular status. Whilst these methods work inside living cells and hence enable a more or less continuous monitoring under the microscope and, quantitatively, by flow cytometry and in a fluorescent plate reader, other methods require the opening of the cell in order to “titrate” the total thiol content or to estimate the concentration of specific proteins by Western Blot. These cell based analytical methods looking at specific events at a time are now joined—and more and more often preceded—by methods attempting to “illuminate” the entire cell, such as methods considering overall gene expression (e.g., levels of mRNA), changes to the proteome (e.g., multidimensional gel electrophoresis coupled to mass spectrometry) or, as in the case of chemogenetic screening, resistance and sensitivity of a wide range of specific mutants. As numerous redox sensitive fluorescent dyes have entered the market during the last decade, and various yeast mutants lacking specific redox proteins and enzymes are easily available from EUROSCARF, this whole cell-based approach is particularly fruitful in studying the mode of action of redox a*c*tive natural products.

Ultimately, whilst redox active secondary metabolites have been around us for centuries, and most of us have already consumed some of them today, research in this area has changed dramatically during the last couple of years, conceptually as well as methodically. Crude plant extracts have given way to pure and often chemically modified compounds and nanoparticles, the notion of antioxidants has been refined to pay dues to complex redox modulation and concepts such as the “cellular thiolstat” have emerged. Today, sophisticated analytical techniques enable us to analyze complex samples, to purify and identify complicated natural molecules, to screen vast libraries of natural compounds and to follow the events such compounds trigger, influence or inhibit inside the living cell via “intracellular diagnostics”. This, in turn, has moved the field forward, from the traditional antioxidant fruit juice to new leads in nutrition and the development of agents useful in therapy, agriculture and cosmetics. This Special Issue provides a glimpse of all that, and naturally also some fascinating reading. Enjoy!
